# 3-Hydroxypyrimidine-2, 4-dione Derivatives as HIV Reverse Transcriptase-Associated RNase H Inhibitors: QSAR Analysis and Molecular Docking Studies

**DOI:** 10.22037/ijpr.2020.1101004

**Published:** 2020

**Authors:** Azar Mostoufi, Narges Chamkouri, Samaneh Kordrostami, Elham Alghasibabaahmadi, Ayyub Mojaddami

**Affiliations:** a *Toxicology Research Center, School of Pharmacy, Ahvaz Jundishapur University of Medical Sciences, Ahvaz, Iran. *; b *Department of Medicinal Chemistry, School of Pharmacy, Ahvaz Jundishapur University of Medical Sciences, Ahvaz, Iran. *; c *Abadan School of Medical Sciences, Abadan, Iran.*

**Keywords:** QSAR, Molecular docking, Reverse transcriptase, AIDS, Antiviral Agents

## Abstract

AIDS, as a lethal disease, is caused by infection with the HIV virus that affects millions of people. Three essential enzymes should be encoded for replication of HIV virus: protease, integrase and reverse transcriptase (RT). RT has two different activities including DNA polymerase and ribonuclease H (RNase H). However, all of the marketed RT inhibitors target only the DNA polymerase activity. Therefore, ribonuclease H activity may serve as a new target for drug discovery. In the present study, a series of 3-Hydroxypyrimidine-2, 4-dione derivatives as potent RT-associated RNase H inhibitors were applied to QSAR analysis. Two methods including multiple linear regressions (MLR) and partial least squared based on genetic algorithm (GA-PLS) were utilized to find the relationship between the structural feathers and inhibitory activities of these compounds. The best multiple linear regression equation was generated by GA-PLS method. A combination of 2D autocorrelations, topological, atom-centered, and geometrical descriptors were selected by GA-PLS as they had more effects on the inhibitory activity. Then, the molecular docking studies were carried out. The results showed that the important amino acids inside the active site of the enzyme responsible for essential interactions were Gln475, Asp549, Tyr501, Ser515, Trp534, Asp493, Tyr472, and Gln480 which took part in hydrogen bond formation. Furthermore, docking energy was plotted against pIC_50_ predicted by GA-PLS method. The result showed that there is a good correlation with R^2^=0.71. Consequently, these findings suggest that the better method, GA-PLS, could be applied to design new compounds and predict their inhibitory activity.

## Introduction

Acquired immune deficiency syndrome (AIDS) is an important health issue around the world. Based on the UNSAIDS report, in 2015 more than 36.7 million people are infected with HIV-1virus and 2.1 million new HIV infections worldwide add up to a total of 36.7 million people living with HIV (1). Thus, the development of novel anti HIV-1 virus drugs is one of the most important goals in medicinal chemistry. 

There are many targets for the control of HIV progression: the reverse transcriptase (RT) enzyme which reverse transcript of viral RNA into viral DNA, integrase enzyme that integrates viral DNA with host DNA, and the protease enzyme which maturates new viral protein (2). RT consists of two catalytic domains:1) the DNA polymerase domain that carries out both RNA-dependent and DNA-dependent viral DNA polymerization and 2) RNase H domain which selectively removes the RNA strand from the RNA/DNA hybrid during reverse transcription process(3).

Most of antiviral drugs used for the treatment of HIV infection are RT inhibitors that particularly affect on the polymerase domain (2). Due to the increase in resistance to current antiviral drugs (4), new target sites within the HIV replication process have recently received more attention (5, 6). One such new target is the inhibition of RT-associated RNase H activity (7). Thus, the development of the RT-associated RNase H inhibitors is a useful strategy to develop effective medications for the treatment of HIV infected patients.

Nowadays, quantitative structure-activity and relationships (QSAR), as a computational method, is particularly used in the drug design process. It is a very useful method to show the quantitative relationship between the biological activities of a particular compound and its structural and physicochemical features (8). Molecular docking simulations, as another drug design methodology, have been used to offer more information about the interaction between molecule and the active site of target.

In this study, QSAR and molecular docking simulations were performed for a series of 3-Hydroxypyrimidine-2, 4-dione derivatives as HIV RT-associated RNase H inhibitors which were designed and synthesized by Z. Wang *et al* (3). In effect, two QSAR models were applied to find the relationship between the structural features and HIV RT-associated RNase H inhibitory activity of these studied compounds. These models included: (i) multiple linear regression (MLR) (ii) partial least squared combined with genetic algorithm for variable selection (GA-PLS). It should be also noted that a validated molecular docking simulation study was also carried out on both datasets and the designed compounds to find out the molecular binding interaction of these compounds with the active site of target. The findings of this study were expected to contribute to understanding the structure-activity relationships of the studied molecules and be used for the design of novel and potent compounds with high HIV RT-associated RNase H inhibitory activity. 

## Experimental

*Data set*

For this QSAR study, a data set consisting of sixty one 3-Hydroxypyrimidine-2, 4-dione derivatives as HIV RT-associated RNase H Inhibitors was selected (3). The structural features and biological activities of these derivatives are listed in Table 1. The biological activities were reported as IC_50_ values and converted to logarithmic scale (IC_50_) and finally used for the QSAR analysis.

*Molecular descriptors*

The two dimensional structures of the ligands were constructed using ChemBioDraw 12.0 software. For minimization energy, the ligands were subjected to minimization procedures by means of an *in house* TCL script using Hyperchem (Version 8, Hypercube Inc., Gainesville, FL, USA). Each ligand was optimized with two minimization methods, first molecular mechanics (MM+) and, then, quantum based semi-empirical method (AM1) using Hyperchem package. Large number of molecular descriptors was calculated using Hyperchem and Dragon package (9). Hyperchem Software was applied to calculate some chemical parameters including molecular volume (V), molecular surface area (SA), hydrophobicity (LogP), hydration energy (HE), and molecular polarizability (MP). The different topological, geometrical, charge, empirical, constitutional, 2D autocorrelations, aromaticity indices, atom-centered fragments, and functional groups descriptors for each molecule were calculated using Dragon 5.0 software. The brief description of some of them is listed in Table 2.

*Model development*

The calculated descriptors were collected in a data matrix whose number of rows and columns were the number of molecules and descriptors, respectively. Two different regression methods were used to construct QSAR equations: (i) simple multiple linear regression with stepwise variable selection (MLR) and (ii) Genetic algorithm–partial least squares (GA-PLS). These already mentioned methods are well applied in the QSAR studies (10, 11).

In the present study, MLR with stepwise selection and elimination of variables was applied for developing QSAR models using SPSS software (version 21; SPSS Inc., IBM, Chicago, IL, USA). The resulted models were validated by leave-one-out cross-validation procedure to check their predictability and robustness using MATLAB 2015 software (version 8.5; Math work Inc., Natick, MA, USA). 

The partial least square (PLS) regression method was applied to the NIPALS-based algorithm existed in the chemometrics toolbox of MATLAB software. Leave-one-out cross-validation procedure was used to obtain the optimum number of factors based on the Haaland and Thomas F-ratio criterion (12, 13). The MATLAB PLS toolbox developed by eigenvector company was used for PLS and GA modeling. All calculations were run on a core i7 personal computer (CPU at 6 MB) with Windows 7 operating system.

*Model validation*

Statistical parameters such as standard error of regression (SE), correlation coefficient (R^2^), variance ratio (F) at specified degrees of freedom, leave-one-out cross-validation correlation coefficient (Q^2^), root mean square error of cross-validation (RMScv) and double cross validation (Cvcv) were employed to calculate the validity of regression equation. In order to test the developed model performances, 20% of the molecules were selected as test set molecules. The predictive value of a QSAR model that was not taken into account during the development process of the model should be tested on an external set of data. The QSAR model was developed in more than three data sets and the best equations were selected as the best model. 

*Applicability domain*

Before using a QSAR for screening chemicals, its domain of application must be defined and predictions for only those chemicals that fall in this domain may be considered reliable. The applicability domain is evaluated by the leverage values for each compound. A Williams plot (the plot of standardized residuals versus leverage values (h)) can then be used for an immediate and simple graphical detection of both the response outliers (Y outliers) and structurally influential chemicals (X outliers) in our model. In this graph, the applicability domain is established inside a squared area within ±x (standard deviations) and a leverage threshold h*. 

The threshold h* is generally fixed at 3(k + 1) ⁄n (k is the number of model parameters and n is the number of training set compounds), whereas x = 2 or 3. Prediction must be considered unreliable for compounds with a high leverage value (h > h*). A leverage greater than warning leverage h* means that the predicted response is the result of substantial extrapolation of the model and, therefore, may not be reliable (14).

*Docking procedure*

The docking studies were carried out by means of an *in house* batch script (DOCKFACE) (15, 16) of AutoDock 4.2. For docking procedure, each ligand was optimized with MM^+ ^and AM1 minimization method using HyperChem 8. Then, the partial charges of atoms were calculated using Gasteiger-Marsili procedure implemented in the AutoDock Tools package (17). Non-polar hydrogens of compounds were merged, and then rotatable bonds were assigned. The output structures were converted to PDBQT using MGLtools 1.5.6 (18). 

The three dimensional crystal structure of HIV RT-associated RNase H (PDB ID: 1hrh) was retrieved from protein data bank (http://www.rcsb.org/pdb/home/home.do). All water molecules were removed and missing hydrogens were added. Then, after determining the Kollman united atom charges, non-polar hydrogens were merged into their corresponding carbons using AutoDock Tools (19). Among the three different search algorithms performed by AutoDock 4.2, the commonly used Lamarckian Genetic Algorithm (LGA) was applied (20). Finally, the PDBQT file of enzyme was obtained using MGLTOOLS 1.5.6.

For Lamarckian GA, a maximum number of 2,500,000 energy evaluations, 27000 maximum generations; 150 population sizes, a gene mutation rate of 0.02; and a crossover rate of 0.8 were applied. The grid maps of the receptors were calculated using AutoGrid tools of AutoDock 4.2. The size of grid was set in a way to include not only the active site, but also the considerable portions of the encircling surface. A grid box of 68×60×70 points in x, y, and z directions was built and centered on the center of the ligand in the complex with a spacing of 0.375 Å. Number of points in x, y and z was -10.012, 20.681 and 45.166, respectively. AutoDock Tools was employed to produce both grid and docking parameter files i.e. gpf and dpf.

In the validity evaluation step of docking process, SMIL format of 17 active ligands and 66 inactive decoys were extracted from ChEMBL database (21). 3D generation of these structures as mol2 format was generated using openbabell software. After docking of the active ligands and inactive decoys based on the applied docking procedure for 3-Hydroxypyrimidine-2, 4-dione derivatives, the area under the curve (AUC) for receiver operating characteristic (ROC) plot was calculated for active ligands and decoys using our application (22). 

Autodock tools program (ADT, Version 1.5.6) and VMD, were applied to show the Ligand-receptor interactions of docking results (23). This software visualizes hydr-ogen bonding, π-aren cation and aren-H as well as hydrophobic interactions which are established through the docking procedure. In addition, the docking energy was plotted versus pIC_50_ predicted by GA-PLS method to obtain their correlation (Figure 5).

## Results and Discussion

In this study, we developed a validated QSAR study using a combination of chemical, electronic and substituent constant, to explore the structural parameters affecting 3-Hydroxypyrimidine-2, 4-dione derivatives as HIV RT-associated RNase H inhibitors. Among the different chemometrics tools available for modeling the relationship between the biological activity and molecular descriptors, two methods (i.e. stepwise MLR, and GA-PLS) were applied.

*MLR *

In the first step, separate stepwise selection-based MLR analyses were performed using different types of descriptors, and then, an MLR equation was obtained utilizing the pool of all calculated descriptors. The results are listed in Table 3. 

To validate the resulted QSAR equations, the statistical parameters calculated for this enzyme, such as R^2^, correlation coefficient (R^2^p) of test set, SE, F at specified degrees of freedom, Q^2^, Cvcv and RMScv were used and are represented in Table 3. Equation 1 (in Table 3) was selected as the best equation in the MLR model. For the studied compounds the HIV RT-associated RNase H inhibitory activity affected by the selected variables in Table 3, included topological (X4A and PCR), 2D autocorrelations (GATS4e, ATS8e, MATS2e and MATS8e), and atom-centered (N-075) descriptors, respectively. 

*GA-PLS*

In PLS analysis, there were data pre-processing steps before obtaining the final statistical QSAR relation, included decomposing of the descriptors data matrix to orthogonal matrices with an inner relationship between the dependent and independent variables. Therefore, the problem of multicolinearity among the descriptors is omitted by PLS analysis. This modeling method coincides with noisy data better than MLR method because a minimal number of latent variables are used for modeling in PLS. The final equation of GA-PLS model is represented in Table 3. 

As shown in the Table 3, a combination of 2D autocorrelations (GTS4e, MATS8v, MATS7v, MATS6e, ATS7v and MATS2e), topological (STN), atom-centered (C-024), and geometrical (TIE) descriptors have been selected by GA-PLS that have more effect on HIV RT-associated RNase H inhibitory activity. In this Table, Eq. 2 with high statistical quality parameters was obtained from the pool of the calculated descriptors (i.e., R^2^ = 0.89 and Q^2^ = 0.73) and, the R^2^_P_ value for the test set was found to be 0.81. These results showed that both QSAR models were not obtained by chance and greater statistical parameters indicated that the best set of calculated descriptors was selected by GA-PLS. 

**Figure 1:**
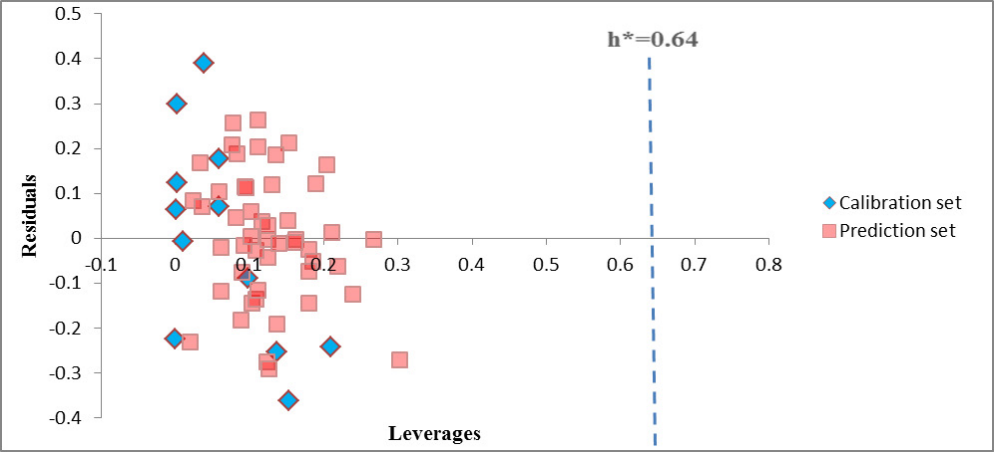
Williams plot for the calibration set and external prediction set for HIV Reverse Transcriptase-Associated RNase H inhibition of studied compounds

The Williams plot of the GA-PLS model of HIV RT-associated RNase H (Figure 1) for the whole data set, indicated that none of the compounds were identified as an obvious outlier for these activity, because of all the compounds had leverage (h) values less than the threshold leverages (h*). The warning leverage (h*) obtained for this enzyme was 0.64. The compounds that had a standardized residual more than three times of the standard deviation units were considered to be outliers. For both the training set and prediction set of targets, the presented model agrees with the high quality parameters with good fitting power and the capability of assessing external data. Moreover, almost all of the compounds were within the applicability domain of the proposed model and were evaluated accurately. 

**Figure 2:**
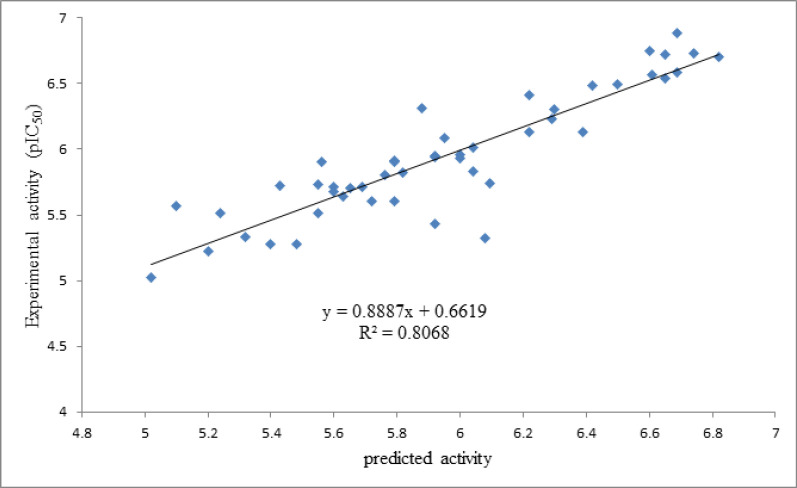
Plots of cross-validated predicted values of HIV Reverse Transcriptase-Associated RNase H inhibitory activity by GA-PLS against the experimental values

As it is shown in Figure 2, the predicted activity data are plotted versus the experimental activities by considering the cross-validated prediction results. The least scattering of data was obtained from GA-PLS, and the high regression ratio (R^2 ^= 0.80) in this plot indicates the high performance of the GA-PLS equation to predict this activity.

*Docking Studies*

In this study, molecular docking simulations were carried out on 61 compounds of dataset, to find out the interactions between HIV RT-associated RNase H target and their inhibitor compounds. The results obtained from this part of the study gave some insight into their molecular binding mode.

**Figure 3:**
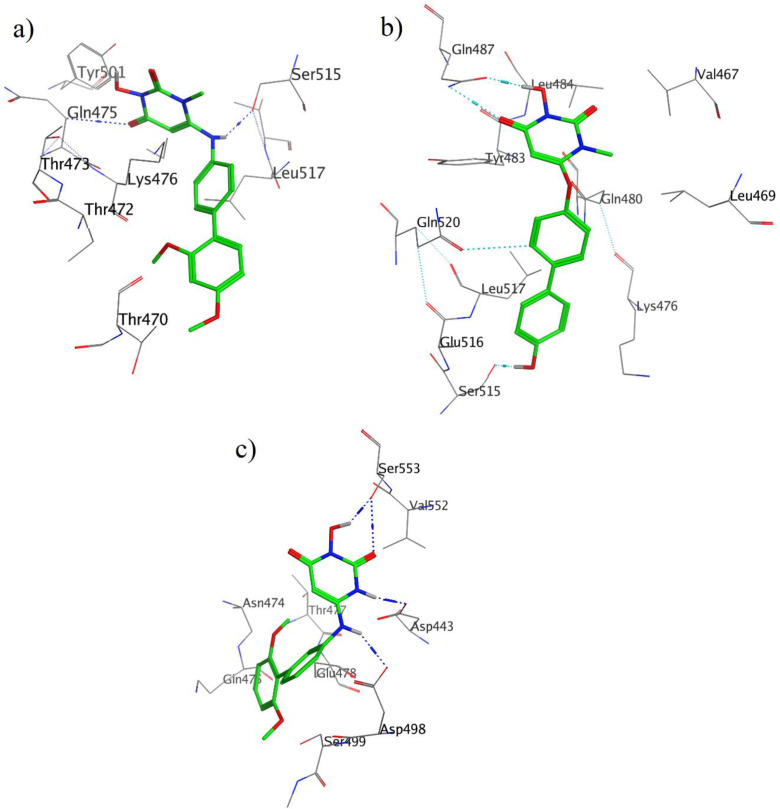
3D of ligand-receptor interactions for **13** (a), **30 **(b) and **59 **(c) in the active site of HIV RT-associated RNase H

Moreover, the calculated free binding energy values (ΔG_bind_, kcal/mol) for the best position of the docked compounds and the corresponding favorable interactions with the key amino acid residues at the active site of enzyme are summarized in Table 1 and Figure 3a-c.

As it is shown in Table 1, the ΔG_bind_ values of the best docked poses of all compounds range from -5.03 to -7.39 Kcal.mol^-1^ and the **20** has the highest binding energy. 

 It should be noted that the important amino acids inside the active site of the enzyme responsible for the essential interactions are Gln475, Asp549, Tyr501, Ser515, Trp534, Asp493, Tyr472, and Gln480 which take part in hydrogen bond formation with different parts of the studied molecules including carbonyl, hydroxyl, and amine moieties. For more details, the interactions of the three compounds in the active site of enzyme are explained below.

As indicated in Figure 3a, two hydrogen bonds were observed; one hydrogen bond between Glu475 and carbonyl moiety, and the other one was one between Ser515 and amine moiety. The Lys 476 residue interacts with the 3-Hydroxypyrimidine-2, 4-dione ring of compound **13** through Aren-H interactions. 

Binding mode of compound **30 **with HIV RT-associated RNase H (Figure 3b) shows that, 3-Hydroxypyrimidine-2, 4-dione ring is involved in two acceptor and donor hydrogen bonds with Gln478. The donor hydrogen bond between hydroxyl group of this ring and carbonyl group of Gln478 residue, while the acceptor one between carbonyl group of ligand and NH of this residue. There is also a hydrogen bond interaction between hydroxynaphtyl group of ligand and carbonyl group of Ser515 residue.

As it was shown in Figure 3c, ligand **59 **interacts via hydrogen bonds through many parts of its molecule, two hydrogen bonds form between carbonyl and hydroxyl groups of ligand and Ser553 residue. In addition to these interactions, NH of Hydroxypyrimidine-2, 4-dione ring and NH of bridge can form a hydrogen bond with Asp443 and Asp498, respectively.

**Figure 4:**
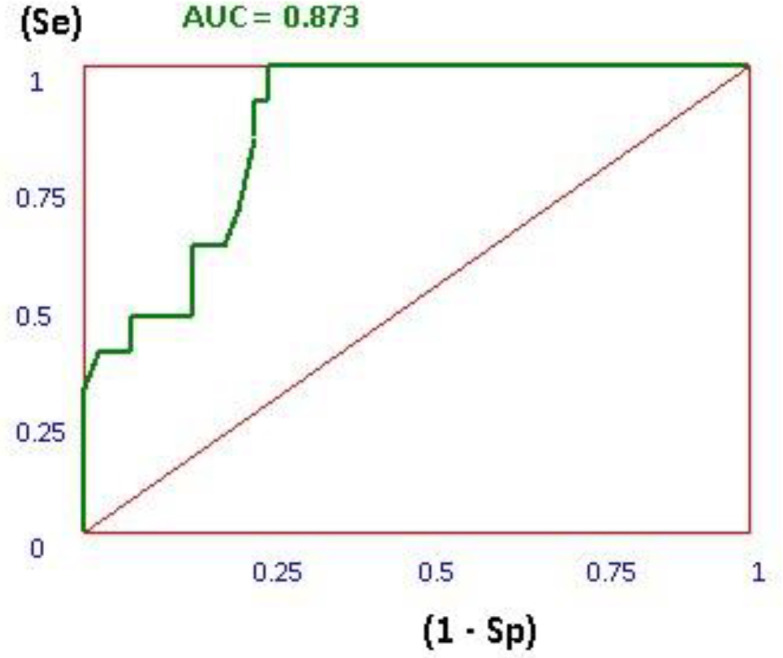
Receiver operating characteristic (ROC) diagram for HIV RT-associated RNase H

Thus, the docking method should be able to distinguish between ligands and decoys. In this plot the true positive rate (sensitivity) versus the false positive rate (1 - specificity) at various threshold settings. The ROC curve is, thus, the sensitivity as a function of 1 - specificity. The more AUC for ROC value means that the docking protocol is more able to distinguish between active ligands and decoys. As it is shown in Figure 4, the AUC of 0.828 showed that the applied docking method was a validated protocol.

**Figure 5:**
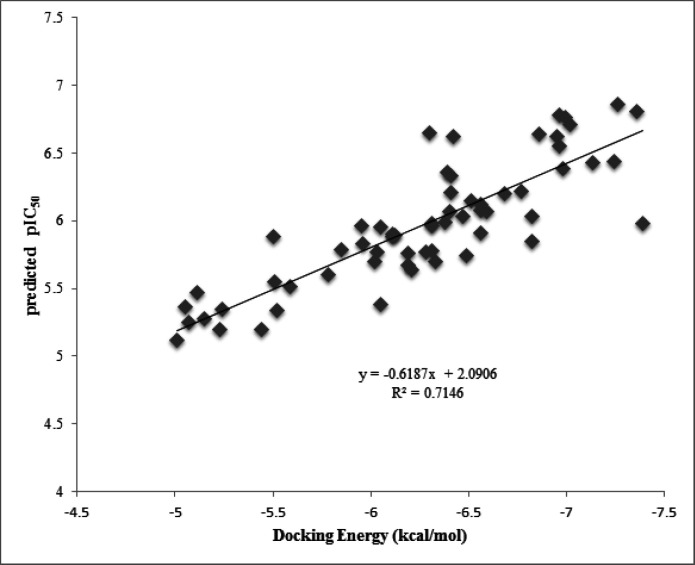
Plot of the docking energy versus calculated pIC_50_ from GA-PLS method

Furthermore, the plot of the docking energy versus predicted pIC50 values reported in Figure 5, indicated that there is good correlation between these two variables with R2=0.71. This result supports this idea that the more docking energy there was, the more interactions were observed.

**Table 1:** Chemical structure of the 3-Hydroxypyrimidine-2,4-dione derivatives used in this study, experimental and cross-validated predicted activity (by GA-PLS) for HIV RT-associated RNase H inhibition as well as their docking binding energy

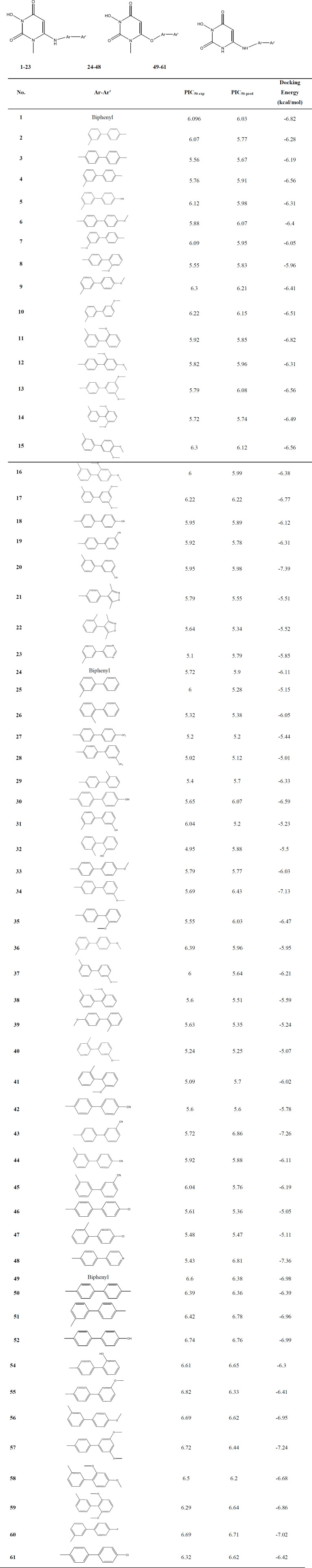

**Table 2:** Definitions of molecular descriptors present in the MLR and GA-PLS models

Descriptors	Brief description
**GATS7v**	Geary autocorrelation - lag 7 / weighted by atomic van der Waals volumes
**GATS4p**	Geary autocorrelation - lag 4 / weighted by atomic polarizabilities
**ATS7v**	Broto-Moreau autocorrelation of a topological structure - lag 7 / weighted by atomic van der Waals volumes
**GATS8v**	Geary autocorrelation - lag 8 / weighted by atomic van der Waals volumes
**MATS7p**	Moran autocorrelation - lag 7 / weighted by atomic polarizabilities
**GATS8e**	Geary autocorrelation - lag 8 / weighted by atomic Sanderson electronegativities
**X4A**	average connectivity index of order 4 Connectivity indices
**PCR**	ratio of multiple path count over path coun Walk and path counts
**C-024**	R--CR—R
**STN**	Sum of tN E-states Atom-type E-state indices
**TIE**	E-state topological parameter Topological indices

**Table 3:** The results of different QSAR models with different type of dependent variables for HIV Reverse Transcriptase-Associated RNase H

**Model**	**Eq.no.**	**MLR Equation**	**n** ^1^	**R** ^2^_c_	**Q** ^2^	**Rmscv**	**Cvcv**	**F**	**SE**	**R** ^2 ^_p_
MLR	1	**pIC50** = -0.667GATs4e (±0.091) + 5.863ATS8e (±0.886) -1.881MATs2e (±0.325) + 45.404 X4A(±16.148) -0.556 N_075 (±0.151) + 89.303GTS4m (±29.810) +0.518MATS8e (±0.2)-0.016PCR (±0.007) +1.268 (±2.022)	49	0.85	0.71	0.37	4.57	64.1	0.12	0.76
GA-PLS	2	**pIC50** = -1.022GATS4e(±0.083) + 56.459X4A (±15.440)-1.849 MATS2e (±0.289) +1.793MATS7v (±0.386) -1.186MATS6e (±0.418) + 4.803ATS7v (±0.827) -4.675 STN (±1.195) + 0.125C_024 (±0.044) + 0.001 TIE (±0)+23.443 (±6.466)	49	0.89	0.73	0.33	4.26	70.8	0.07	0.81

^1 ^Number of molecules of training set used to derive the QSAR models

R^2^: Regression Coefficient for Calibration set

Q^2^: Regression Coefficient for Leave One Out Cross Validation

RMS_cv_: Root Mean Square Error of cross validation

R^2^p: Regression Coefficient for prediction set

## Conclusion

Two methods of QSAR, MLR, and GA-PLS, were employed to obtain the quantitative relationships between molecular feathers of 3-Hydroxypyrimidine-2,4-dione derivatives and HIV RT-associated RNase H inhibitory activity. The cross-validation, the root mean square error of prediction (RMSEP), root mean square error of cross-validation (RMSECV) and Y-randomization were utilized to verify the reliability, accuracy, and predictability of the proposed models. Based on the statistical parameters, the GA-PLS method is better than MLR method for the prediction of the HIV RT-associated RNase H inhibitory activity. The results obtained from the validated molecular docking studies gave some insight into molecular binding mode, and the importance of amino acids in the active site of enzyme as they have significant roles in the inhibitory activity. There was a good correlation between the docking energy and pIC_50 _predicted by GA-PLS method. Finally, these finding can be used to design more active HIV RT-associated RNase H Inhibitors.
